# The Book World of Medicine and Science

**Published:** 1898-10-01

**Authors:** 


### The Book World of Medicine and Science.

The People's Guide to the Workman's Compensation Act. By R. M. Minton Senhouse and G. F. Emery, LL.M. Pp. 23. (London: Bemrose and Sons, Limited. 1898. Price 1d.)

Handbook to the Workman's Compensation Act, 1897. By M. Roberts Jones. Third edition. Pp. 79. (Cardiff: *The Western Mail.* 1898. Price 2s.)

We have before us two popular guides to the Workman's Compensation Act of 1897. The first—by Messrs. Senhouse and Emery—is of such size that it may be slipped in the waistcoat pocket, and sets forth clearly and shortly the scope of the Act, the compensation proposed, and the procedure necessary for its recovery. It is, in fact, just such a little book as will prove useful to all workmen likely to be affected by the provisions of the Act.

Mr. Robert Jones's book is more ambitious. In the first place it gives, with marginal notes, the complete text of the Act and of the schedules relating to compensation and arbitration. Detailed consideration is given to the subject of accidental injuries, wilful misconduct, and liability of the workmen. Judicial decisions are freely quoted and the whole matter made as clear as is at present possible. One interesting chapter relates to voluntary schemes of compensation and furnishes specimen rules extracted from agreements which have already been completed between employers and workmen. Lastly, appendices are given in which references in the Act to other statutes are elucidated, and various forms for notices of claim, &c., are suggested. The subject matter of the book is relieved by free quotation from speeches of ministers, and its use is facilitated by an excellent index. As the author says, years will elapse before the Act is clearly understood by lawyers and laity. In the meantime Mr. Roberts Jones's excellent handbook will doubtless meet with much popular favour.

The Mineral Waters and Health Resorts of Europe. By Hermann Weber, M.D., F.R.C.P., and F. Parkes Weber, M.D., F.R.C.P. (London: Smith, Elder, and Co. 1898. Price 10s. 6d.)

This book is a revised and enlarged edition of "The Spas and Mineral Waters of Europe." The revision has been carefully done, and much new matter has been interpolated. At first sight it might appear an easy matter to write a book on watering-places, and to those contented with taking a superficial view of things, perhaps it might be. Anyone, however, who will read this book carefully, as we have done, will recognise how far from superficial is the view taken of the very wide subject with which it deals, and will see how large and carefully-collated an experience has been condensed into its pages. The book begins with a chapter on "Hydrotherapeutics and the Effects of Plain Water." Then we find a description of the constituents of mineral waters, their classification, and their action. But people who go to spas do not drink and bathe only. They undergo various kinds of exercises and massage, which in some cases form a very important part of the treatment, and all these proceedings are carefully described. A general sketch is then given of the daily life at a typical spa, details being entered into with regard to the seasons at which the different "cures" are undertaken, and the time which they require. And after this comes a careful appreciation of the various exercises and forms of massage which form so important a part of the treatment given at certain spas. The mass of the book is devoted to a description of the various health resorts which are available in Europe for the treatment of all kinds of disease. The spas are classified according to the nature of their waters, the character of their climates, and the "cures" and special methods of treatment for which they are more particularly resorted to. This portion of the book tends, of course, towards the dictionary type, but it is saved from dulness by its arrangement. Under each heading, such as "Iron Waters," "Sulphur Waters," "Arsenical Waters," &c., a more or less detailed description is given of one or two typical examples, and then the rest are mentioned very shortly, so that the first part of each chapter gives a good description of the action of the class of waters of or climate with which it deals, and this is followed by a list of the other places where the same sort of treatment can be obtained. This is undoubtedly the pleasantest arrangement for reading, although perhaps for reference the alphabetical arrangement might be more convenient. The chapter on grape cures, milk and whey cures, sanatoria for dietetic and special methods of treatment, and sanatoria for the treatment of phthisis is a most useful one, dealing as it does with a series of subjects on which it is not easy to obtain recent and reliable information. Altogether the book is one that can be read with great interest. It is full of valuable hints in regard to hydro-therapeutics and the climatic treatment of disease, besides being an authoritative dictionary of health resorts.

Buxton: Its Baths and Climate. By Samuel Hyde, M.D. (Manchester and London: John Heywood. London: Simpkin, Marshall, and Co. 1889. Pp. 103.)

As nine years have elapsed since the first appearance of this work, the author has found it necessary to revise and add to this second edition. The chapter on "The Medical Action and Uses of the Waters and Baths" has been entirely rewritten. New chapters on Buxton as a resort for consumptives and as an educational centre have been added. There is a chapter on the various kinds of baths, and a full discussion of the whole question of the thermal and chalybeate waters and their actions and modes of use, as well as chapters on the climate and life generally at Buxton. Various important accessory methods of treatment, such as diet, massage, &c., are discussed. Dr. Hyde's book is already too well known to need any introduction, and the present edition will be found useful to medical practitioners, not only as regards the details, but as regards the general principles ruling the selection or non-selection of Buxton as a health resort for their various patients.

---

We regret that in a review of "A Dictionary of Diseases and their Treatment," which appeared last week, its publication was by an error attributed to the office of the *Chemist and Druggist.*

